# Factors and pathways influencing sugar-sweetened beverage consumption among children in middle childhood: a cross-sectional survey of 1,127 third-grade students in Beijing

**DOI:** 10.3389/fpubh.2026.1817212

**Published:** 2026-05-20

**Authors:** Lulu Meng, Wenjia Li, Yiran Li, Siyu Liang, Manning Wang, Liyu Huang, Ruoxiang Cao

**Affiliations:** 1 Office of Research and Teaching Administration, Beijing Center for Disease Prevention and Control, Beijing, China; 2 Xingtai Xindu District Center for Disease Control and Prevention, Xingtai, Hebei, China; 3 School of Public Health, Hebei Medical University, Shijiazhuang, Hebei, China; 4 School of Public Health, Capital Medical University, Beijing, China; 5 Institute of Nutrition and Food Hygiene, Beijing Center for Disease Prevention and Control, Beijing, China

**Keywords:** behavior, family, middle childhood, peer, social-ecological model, sugar-sweetened beverages

## Abstract

**Background:**

Sugar-sweetened beverage (SSB) consumption among children represents a significant risk factor for health problems, including obesity and dental caries. Children aged 8 ~ 9 years are in a critical developmental period for behavioral habit formation; however, the mechanisms underlying these behaviors require deeper investigation. Grounded in social-ecological theory, this study systematically explores the associations between multilevel factors—encompassing individual, interpersonal, environmental, and awareness of health labeling—and children’s SSB consumption.

**Methods:**

We conducted a cross-sectional survey from October to November 2024 among 1,127 students from six elementary schools in Beijing. Using multistage cluster random sampling, we administered standardized questionnaires to collect data on SSB consumption and its multilevel influencing factors. Structural equation modeling was employed to analyze the pathways linking individual, interpersonal, environmental, and health labeling factors to SSB consumption.

**Results:**

The prevalence of SSB consumption in the past week among the study population was 81.90% (923/1,127). The structural equation model demonstrated acceptable fit. Students’ SSB consumption was significantly predicted by caregivers’ SSB consumption (*β* = 0.378), peer influence (*β* = 0.254), home SSB storage (*β* = 0.230), caregivers’ rewards (*β* = 0.096), teachers’ rewards (*β* = 0.081), and students’ attitudes (*β* = −0.148), all *p* < 0.05. Students’ attitudes were positively influenced by awareness of health labeling (*β* = 0.778), students’ knowledge level (*β* = 0.427), and caregivers’ attitudes (*β* = 0.183), all *p* < 0.001. Health education courses positively predicted students’ knowledge level (*β* = 0.188, *p* < 0.001). Caregivers’ SSB consumption was positively associated with convenience store access (*β* = 0.148, *p* < 0.001) and home SSB storage (*β* = 0.106, *p* < 0.01). Significant indirect effects were observed via students’ attitudes and caregivers’ SSB consumption. The model explained 35.2% of the variance in students’ SSB consumption.

**Conclusion:**

SSB consumption is highly prevalent among elementary school students in Beijing, with interpersonal factors (caregivers and peers) exerting the most prominent influence. Future interventions should establish an integrated, comprehensive system coordinating family, school, and community efforts to reduce children’s SSB consumption and promote healthy behavioral patterns.

## 1 Introduction

Excessive consumption of sugar-sweetened beverages (SSBs) among children has emerged as a critical global public health challenge. Extensive epidemiological and evidence-based medical research demonstrates direct associations between SSB consumption and childhood obesity, dental caries, and type 2 diabetes, with long-term implications for cardiovascular and metabolic health extending into adulthood (1–5). This escalating health concern has prompted international policy responses. For example, the U.S. Department of Health and Human Services (HHS) and the U.S. Department of Agriculture (USDA) jointly released the Dietary Guidelines for Americans, 2025–2030 on January 7, 2026, which explicitly recommends avoiding SSB consumption (6). In China, this issue has become particularly acute: SSB consumption among residents has increased rapidly since 2000 (7), with recent data indicating that daily SSB intake among Chinese urban residents substantially exceeds the global average, suggesting an elevated disease burden and heightened health risks (8, 9).

Many megacities worldwide have prioritized SSB control on their policy agendas, implementing comprehensive frameworks that include taxation, sales restrictions, mandatory nutrition labeling, and advertising regulations (10–12). China’s response strategies, however, have focused primarily on health education and sales restrictions. Shanghai and Shenzhen have both issued health labeling standards and guidelines (13, 14), while Beijing has adopted measures including restrictions on high-sugar beverage sales on campus and enhanced health education. Despite these efforts, a comprehensive, multilevel governance framework integrating family, school, community, and market sectors across all relevant settings has yet to be established.

SSB consumption behavior is shaped by multilevel, multidimensional factors. Existing research has predominantly examined single-level influences, such as individual knowledge and attitudes or family environment (15), without systematically quantifying the interactive and combined effects of individual, interpersonal, environmental, and policy factors within an integrated theoretical framework. This gap limits our understanding of behavioral determinants and hinders the development of precisely targeted, multilevel intervention strategies.

The social-ecological model offers essential theoretical support for addressing these challenges by recognizing that individual behaviors are embedded within multiple environmental systems. This framework provides a robust foundation for analyzing the complex interactions among behavioral determinants and designing comprehensive intervention strategies (16–18). Applied to sugar-sweetened beverage consumption, the model demonstrates that behavior is shaped simultaneously by individual factors (e.g., cognition, preferences), interpersonal factors (e.g., family and peer influences), environmental factors (e.g., school policies), and macro-level policy factors (e.g., taxation, advertising regulations) (19). While comprehensive interventions grounded in this model have shown short-term effectiveness in improving adolescent health behaviors (20), structural equation modeling validation using large-scale empirical data remains limited. Specifically, research has not adequately examined how factors at various levels influence consumption behavior through direct and indirect pathways, nor has it clarified their relative contributions and mechanisms of action among urban Chinese children.

Third-grade elementary school students (8 ~ 9 years old) represent a developmental stage known as “Middle Childhood” in developmental psychology. During this period, children experience significant cognitive, social, and emotional transitions, with behavioral patterns shifting from complete family dependence toward increasing influence from peers and school environments (21). Investigating sugar-sweetened beverage consumption behaviors in this population therefore offers a strategic opportunity to capture the critical window for habit formation, systematically examine the interactive influences of multilevel environments—including family, peers, and schools—and establish optimal timing for early, targeted interventions supported by scientific evidence.

Building on this foundation, the present study employs the social-ecological model as a theoretical framework to conduct a cross-sectional survey of elementary school students in Beijing with three primary objectives: (1) to characterize the prevalence of sugar-sweetened beverage consumption in this population; (2) to use structural equation modeling to quantitatively analyze the direct and indirect pathways through which individual, interpersonal, environmental, and health labeling factors influence consumption behaviors, thereby revealing their complex mechanisms of action; (3) to provide empirical, scientific evidence for developing and implementing precise, multilevel comprehensive intervention strategies in megacities such as Beijing, based on the key pathways and nodes identified through our findings.

## 2 Materials and methods

### 2.1 Study population

This cross-sectional study was conducted between October and November 2024. We recruited participants using multistage cluster random sampling. First, we stratified Beijing into three tiers based on administrative planning, economic development, and educational resource distribution: suburban districts, outer urban districts, and central urban districts. We then randomly selected one administrative district from each tier as a survey site and randomly selected two elementary schools from each district. Finally, we enrolled all third-grade students from each sampled school along with one primary caregiver per student. In total, 1,127 students from six elementary schools participated in this study.

The Beijing Center for Disease Prevention and Control Ethics Committee approved this study (BJCDC2024031). All participants and their caregivers provided written informed consent before study participation.

### 2.2 Data collection

This study employed two custom-developed questionnaires: the “Sugar-Sweetened Beverage Consumption Behavior Questionnaire” and the “Sugar-Sweetened Beverage Consumption Family Environment Questionnaire.” These instruments assessed personal demographic information, students’ SSB knowledge level, attitudes, consumption behaviors, and associated influencing factors. Both questionnaires were developed through systematic literature review, followed by multiple rounds of expert consultation and pilot testing. The instruments demonstrated good internal consistency reliability, with Cronbach’s *α* coefficients of 0.81 and 0.75, respectively.

The survey was conducted through standardized on-site administration. Students completed the “Sugar-Sweetened Beverage Consumption Behavior Questionnaire” collectively in their classrooms under the guidance of trained investigators. The “Sugar-Sweetened Beverage Consumption Family Environment Questionnaire” was distributed to students with written instructions for completion by their primary caregiver at home and returned within the designated timeframe.

### 2.3 Measures

#### 2.3.1 Sugar-sweetened beverage consumption

The assessment of students’ SSB consumption was based on self-reported information on the frequency and volume of consumption for both prepackaged and ready-to-drink SSBs during a 7-day period [operational definition is provided in reference (22)]. For the analysis, the consumption rate for each beverage category was expressed in terms of weekly occurrences, and then summed to obtain the cumulative weekly intake frequency. This was subsequently classified into four categories: never (no consumption in the past week), 1–3 times/week, 4–6 times/week, and ≥7 times/week (23).

SSB consumption = ∑(Weekly consumption frequency of prepackaged SSBs+Weekly consumption frequency of ready-to-drink SSBs).

#### 2.3.2 Students’ knowledge and attitudes

Students’ knowledge score was evaluated using ten questions that addressed the definition of SSBs and their associated health hazards, with 1 point awarded for each correct response, yielding a maximum score of 10 points. Knowledge level was classified into three categories based on total scores: poor (≤5 points), needs improvement (6–7 points), and good (≥8 points) (24); a score of ≥8 points was defined as indicating adequate knowledge for calculating the awareness rate (25).

The study used a correspondingly validated Likert-type questionnaire (ranging from 1 for “completely disagree” to 5 for “completely agree”) to measure students’ perception across three dimensions: health beliefs, self-efficacy, and emotional attitudes. The measurement tool consisted of three items for health beliefs, five items for self-efficacy, and three items for emotional attitudes. The composite score for each domain was obtained by summing the participants’ responses; the higher the value, the more the positive orientation. For the analysis, these continuous scores were transformed into binary categories (poor attitude and good attitude) using median cutoffs derived from the reference data (26). A score of 14 or lower for health beliefs, 15 or lower for self-efficacy, and 10 or lower for emotional attitudes was defined as indicative of a “poor attitude.”

#### 2.3.3 Caregivers’ knowledge, attitudes, and behaviors

Caregivers’ SSB knowledge level was evaluated through five questions, yielding a maximum score of 5 points and classified into three categories: poor (1–2 points), needs improvement (3 points), and good (4–5 points) (24); a score of ≥4 points was defined as indicating adequate knowledge (25).

Caregivers’ attitudes toward restricting their children’s intake of unhealthy foods—including SSBs, fried foods, and high-sugar snacks—were assessed using a 5-point Likert scale.

Caregivers’ SSB consumption during the past week was defined consistently with the student consumption behavior criteria described in Section 2.3.1.

Caregivers’ rewards were assessed by measuring the frequency with which caregivers used SSBs as rewards (never/occasionally/often).

#### 2.3.4 Peers’ and teachers’ behaviors

Peer influence was evaluated through three indicators: peers’ SSB consumption (never/occasionally/often), peer-influenced SSB purchase (never/occasionally/often), and SSB sharing with peers (never/occasionally/often).

Teachers’ rewards were assessed by measuring the frequency with which teachers used SSBs as rewards (never/occasionally/often).

#### 2.3.5 Home, school, and community environments

The home environment was evaluated by assessing the frequency of SSB storage in the home (none/occasionally/frequently).

The school environment was measured by determining the frequency of health education courses (none/occasionally/frequently).

The community environment was assessed by evaluating the convenience of obtaining SSBs from a supermarket, a convenience store, a beverage vending machine, a traditional market, or a restaurant located within a 500-meter walking distance from the family’s residence (convenient/inconvenient/none).

#### 2.3.6 Awareness of health labeling

This section assessed students’ awareness of health labeling through three items: impact on SSB purchase (buy more, no change, buy less, choose low-sugar or no-sugar beverages, or give up purchasing), whether students would discourage family members/friends from consuming SSBs after viewing the labels (yes/no), and their perception of the importance of such labels for developing healthy eating habits (very meaningful, meaningful, slightly meaningful, or not meaningful).

### 2.4 Structural equation modeling

**Figure 1:**
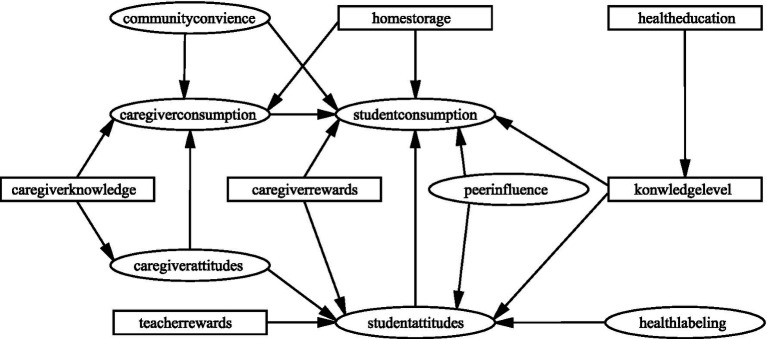
Simplified path diagram illustrating the assumed theoretical structure of the model. Circles represent latent variables, whereas squares represent observed variables.

Using the social-ecological model as a theoretical framework, we constructed a structural equation model with students’ SSB consumption as the dependent variable and factors at the individual, interpersonal, environmental, and policy levels as independent variables (Figure 1). The model incorporated the following levels:

Individual level: students’ knowledge level and attitudes;

Interpersonal level: caregivers’ knowledge level, attitudes, consumption, and rewards; peer influence; teachers’ rewards;

Environmental level: frequency of SSB storage in the home, frequency of health education courses, and convenience of obtaining SSBs from a supermarket, a convenience store, a beverage vending machine, a traditional market, or a restaurant;

Policy level: awareness of health labeling.

The model evaluated both direct and indirect effects of each variable through effect decomposition. Direct effects represent the immediate pathway from independent variables to the dependent variable, whereas indirect effects capture the influence of independent variables on the dependent variable mediated by intermediate variables. All reported path effect values are standardized path coefficients.

### 2.5 Statistical analysis

We performed data cleaning and statistical description of the questionnaire survey data using SPSS 20.0 software. Statistical descriptions included demographic characteristics of the study subjects and frequencies of different types of SSBs. Relationships between students’ SSB consumption and independent variables were assessed using the Kruskal–Wallis test (for comparisons involving one ordinal and one nominal variable) and the linear-by-linear association chi-square test for trend (for two ordinal variables).

We constructed the model using the lavaan package in R 4.5.3. When using WLSMV estimation for ordinal data, traditional bootstrap methods are not supported; Monte Carlo methods are recommended for obtaining confidence intervals for indirect effects (27, 28). Variance inflation factors (VIFs) were calculated to check for multicollinearity, and all values were below 5, indicating no multicollinearity among the measured variables. The proposed model was refined through several cycles of evaluation and adjustment to improve overall fit. Model fit was assessed using multiple indices: χ^2^/df, RMSEA (root mean square error of approximation), GFI (goodness-of-fit index), PGFI (parsimony goodness-of-fit index), TLI (Tucker–Lewis index), and CFI (comparative fit index). The significance level was set at 0.05, with *p* < 0.05 indicating statistical significance.

## 3 Results

### 3.1 Basic characteristics

The sample included students from schools in suburban areas (33.63%, 379 students), outer urban areas (31.32%, 353 students), and central urban areas (35.05%, 395 students). The mean student age was 8.56 ± 0.30 years; boys comprising 50.84% (573 students) and girls 49.16% (554 students) of the sample. Only children accounted for 57.28% (645 students) of participants. Mothers served as the primary caregivers for 69.72% (783 students) of families, and 75.16% (847 students) of primary caregivers held a bachelor’s degree or above (Table 1).

**Table 1:** Frequency of sugar-sweetened beverage consumption among 1,127 students across demographic groups in Beijing.

Variable	Region			Sex		Number of children in household^*^		Primary caregiver type**			Primary caregiver education level		
	Suburban	Outer urban	Central urban	Male	Female	1	≥2	Mother	Father	Grandparents	High school or lower	Associate degree or vocational college	Bachelor’s degree or above
Total (*n* = 1,127)	379 (33.63)	353 (31.32)	395 (35.05)	573 (50.84)	554 (49.16)	645 (57.28)	481 (42.72)	783 (69.72)	326 (29.03)	14 (1.25)	72 (6.39)	208 (18.46)	847 (75.16)
Prepackaged SSBs													
Never	65 (17.15)	92 (26.06)	139 (35.19)	152 (26.53)	144 (25.99)	172 (26.67)	124 (25.78)	210 (26.82)	84 (25.77)	2 (14.29)	9 (12.50)	48 (23.08)	239 (28.22)
1–3 times/week	70 (18.47)	125 (35.41)	129 (32.66)	163 (28.45)	161 (29.06)	203 (31.47)	121 (25.16)	225 (28.74)	90 (27.61)	6 (42.86)	19 (26.39)	61 (29.33)	244 (28.81)
4–6 times/week	58 (15.30)	45 (12.75)	55 (13.92)	85 (14.83)	73 (13.18)	91 (14.11)	67 (13.93)	112 (14.30)	45 (13.80)	1 (7.14)	7 (9.72)	31 (14.90)	120 (14.17)
≥7 times/week	186 (49.08)	91 (25.78)	72 (18.23)	173 (30.19)	176 (31.77)	179 (27.75)	169 (35.14)	236 (30.14)	107 (32.82)	5 (35.71)	37 (51.39)	68 (32.69)	244 (28.81)
*P*-value	<0.001			0.770		0.040		0.660			<0.001		
Ready-to-drink SSBs													
Never	104 (27.44)	160 (45.33)	208 (52.66)	261 (45.55)	211 (38.09)	285 (44.19)	186 (38.67)	333 (42.53)	133 (40.80)	4 (28.57)	20 (27.78)	70 (33.65)	382 (45.10)
1–3 times/week	94 (24.80)	108 (30.59)	111 (28.10)	155 (27.05)	158 (28.52)	178 (27.60)	135 (28.07)	209 (26.69)	95 (29.14)	8 (57.14)	30 (41.67)	66 (31.73)	217 (25.62)
4–6 times/week	87 (22.96)	26 (7.37)	34 (8.61)	68 (11.87)	79 (14.26)	83 (12.87)	64 (13.31)	105 (13.41)	42 (12.88)	0 (00)	10 (13.89)	23 (11.06)	114 (13.46)
≥7 times/week	94 (24.80)	59 (16.71)	42 (10.63)	89 (15.53)	106 (19.13)	99 (15.35)	96 (19.96)	136 (17.37)	56 (17.18)	2 (14.29)	12 (16.67)	49 (23.56)	134 (15.82)
*P*-value	<0.001			0.007		0.027		0.965			0.007		
Total consumption													
Never	45 (11.87)	64 (18.13)	95 (24.05)	110 (19.20)	94 (16.97)	116 (17.98)	88 (18.30)	147 (18.77)	56 (17.18)	1 (7.14)	5 (6.94)	33 (15.87)	166 (19.60)
1–3 times/week	48 (12.66)	99 (28.05)	117 (29.62)	134 (23.39)	130 (23.47)	171 (26.51)	93 (19.33)	177 (22.61)	80 (24.54)	4 (28.57)	16 (22.22)	34 (16.35)	214 (25.27)
4–6 times/week	38 (10.03)	65 (18.41)	68 (17.22)	88 (15.36)	83 (14.98)	107 (16.59)	64 (13.31)	122 (15.58)	47 (14.42)	2 (14.29)	9 (12.50)	39 (18.75)	123 (14.52)
≥7 times/week	248 (65.44)	125 (35.41)	115 (29.11)	241 (42.06)	247 (44.58)	251 (38.91)	236 (49.06)	337 (43.04)	143 (43.87)	7 (50.00)	42 (58.33)	102 (49.04)	344 (40.61)
*P*-value	<0.001			0.321		0.010		0.735			<0.001		

SSB, sugar-sweetened beverage; *: 1 missing, **: 4 missing.

### 3.2 Sugar-sweetened beverage consumption

Among survey participants, 81.90% (923/1,127) reported consuming SSBs, with 73.74% (831/1,127) consuming prepackaged SSBs and 58.12% (655/1,127) consuming ready-to-drink SSBs. Regional analysis of consumption frequency revealed substantial variation in the proportion of students consuming SSBs ≥7 times/week: suburban areas (65.44%), outer urban areas (35.41%), and central urban areas (29.11%) (*p* < 0.001). Non-only children exhibited higher consumption frequency than only children (*p* = 0.010). Students whose primary caregiver had a high school education or lower demonstrated higher consumption frequency (*p* < 0.001). Beyond regional, family size, and caregiver education effects, gender differences emerged specifically in ready-to-drink SSB consumption, with girls consuming more than boys (*p* = 0.007) (Table 1).

### 3.3 Analysis of factors influencing sugar-sweetened beverage consumption

Individual level: There was a significant correlation between students’ SSB consumption and self-efficacy (H = 5.655, *p* = 0.017) and emotional attitudes (H = 9.288, *p* = 0.002), whereas knowledge level and health beliefs showed no significant association (*p* = 0.062 and *p* = 0.171) (Supplementary Table S1).

Interpersonal level: Factors such as caregivers’ knowledge level (χ^2^ = 5.017, *p* = 0.025), caregivers’ SSB consumption (χ^2^ = 61.894, *p* < 0.001), and the use of SSBs as rewards by caregivers and teachers (*p* = 0.001 and *p* = 0.014) were significantly linked to students’ SSB consumption. Additionally, peers’ SSB consumption (χ^2^ = 20.466, *p* < 0.001), peer-influenced SSB purchase (χ^2^ = 66.949, *p* < 0.001), and SSB sharing with peers (χ^2^ = 46.810, *p* < 0.001) were also significantly associated (Supplementary Table S2).

Environmental level: Among environmental factors, frequency of SSB storage in the home (χ^2^ = 49.150, *p* < 0.001) and convenience store access (χ^2^ = 5.792, *p* = 0.016) were significantly related to students’ SSB consumption, while other factors were not statistically significant (*p* > 0.05) (Supplementary Table S3).

Awareness of health labeling: The influence of awareness of health labeling on purchasing SSB (χ^2^ = 42.029, *p* < 0.001), on discouraging family members/friends from consuming SSB (H = 48.124, *p* < 0.001), and on the perceived importance of developing healthy eating habits (χ^2^ = 27.176, *p* < 0.001) were all significantly associated with SSB consumption (Supplementary Table S4).

### 3.4 Structural equation model

The model incorporated six latent variables: students’ SSB consumption, students’ attitudes, caregivers’ SSB consumption, caregivers’ attitudes, peer influence, and awareness of health labeling. The model was developed through iterative specification and refinement guided by theoretical considerations. The final model demonstrated an acceptable fit to the data: χ^2^ = 1052.124, df = 190, χ^2^/df = 5.537. Given the relatively large sample size (*N* = 1,127), the χ^2^ statistic was virtually guaranteed to be significant; therefore, model fit was primarily evaluated based on other fit indices that are less sensitive to sample size. All other fit indices met the recommended thresholds: RMSEA = 0.065, GFI = 0.930, PGFI = 0.669, TLI = 0.940, and CFI = 0.916 (Table 2). The model explained 35.2% of the variance in students’ SSB consumption (*R*^2^ = 0.352).

**Table 2:** Model fit indices for the modified structural equation model.

Goodness-of-fit indices	Standards	Measurement value
χ^2^/*df*	1 ~ 3, 3 ~ 5 acceptable	5.537
RMSEA	<0.05, <0.08 acceptable	0.065
GFI	>0.9, 0.8 ~ 0.9 acceptable	0.930
PGFI	>0.5	0.669
TLI	>0.9, 0.8 ~ 0.9 acceptable	0.940
CFI	>0.9, 0.8 ~ 0.9 acceptable	0.916

RMSEA, root mean square error of approximation; GFI, goodness-of-fit index; PGFI, parsimony goodness-of-fit index; TLI, Tucker–Lewis index; CFI, comparative fit index.

**Figure 2:**
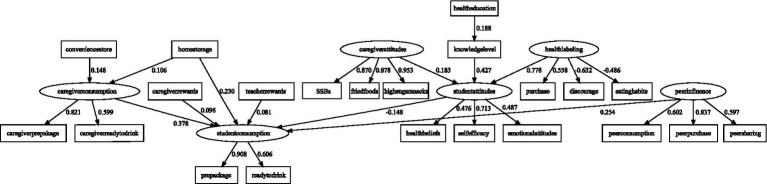
Structural equation model depicting the multilevel factors influencing sugar-sweetened beverage (SSB) consumption. Circles represent latent variables, whereas squares represent observed variables. The numbers adjacent to the arrows represent the standardized path coefficients.

As shown in Table 3 and Figure 2, students’ SSB consumption was positively predicted by caregivers’ SSB consumption (*β* = 0.378, *p* < 0.001), peer influence (*β* = 0.254, *p* < 0.001), home SSB storage (*β* = 0.230, *p* < 0.001), caregivers’ rewards (*β* = 0.096, *p* = 0.008), and teachers’ rewards (*β* = 0.081, *p* = 0.017). In contrast, students’ attitudes were negatively associated with their SSB consumption (*β* = −0.148, *p* = 0.005).

**Table 3:** Test results of the hypothesis.

Path	Non-standardized path coefficient (*b*)	*S. E.*	*C. R.*	*p*-value	Standardized path coefficient (*β*)
Students’ SSB consumption ←					
Caregivers’ SSB consumption	0.432	0.067	6.444	< 0.001	0.378
Peer influence	0.400	0.074	5.434	< 0.001	0.254
Students’ attitudes	−0.296	0.105	−2.810	0.005	−0.148
Teachers’ rewards	0.246	0.103	2.382	0.017	0.081
Home SSB storage	0.314	0.051	6.162	< 0.001	0.230
Caregivers’ rewards	0.168	0.064	2.636	0.008	0.096
Students’ attitudes ←					
Caregivers’ attitudes	0.100	0.029	3.509	< 0.001	0.183
Awareness of health labeling	0.663	0.084	7.937	< 0.001	0.778
Student’s knowledge level	0.200	0.035	5.644	< 0.001	0.427
Student’s knowledge level ←					
Health education courses	0.309	0.073	4.231	< 0.001	0.188
Caregivers’ consumption ←					
Home SSB storage	0.127	0.046	2.787	0.005	0.106
Convenience store access	0.280	0.073	3.836	< 0.001	0.148

SSB, sugar-sweetened beverage; S. E., standard error; C.R., critical ratio.

Students’ attitudes were significantly influenced by the awareness of health labeling (*β* = 0.778, *p* < 0.001), students’ knowledge level (*β* = 0.427, *p* < 0.001), and caregivers’ attitudes (*β* = 0.183, *p* < 0.001). Health education courses positively predicted students’ knowledge level (*β* = 0.188, *p* < 0.001). Caregivers’ SSB consumption was positively associated with convenience store access (*β* = 0.148, *p* < 0.001) and home SSB storage (*β* = 0.106, *p* = 0.005).

As summarized in Table 4, caregivers’ attitudes [*β* = −0.030, 95% CI (−0.054, −0.007)], awareness of health labeling [*β* = −0.196, 95% CI (−0.198, −0.036)], and students’ knowledge level [*β* = −0.059, 95% CI (−0.116, −0.019)] exerted significant indirect effects on students’ SSB consumption via students’ attitudes. Health education courses also showed a significant indirect effect through students’ knowledge level and attitudes [*β* = −0.018, 95% CI (−0.024, −0.003)]. Finally, home SSB storage indirectly influenced students’ SSB consumption via caregivers’ SSB consumption [*β* = 0.055, 95% CI (0.012, 0.072)].

**Table 4:** Indirect effects of predictor variables.

Path	Estimate	95% CI
Caregivers’ attitudes **→** students’ attitudes **→** students’ SSB consumption	−0.030	−0.054, −0.007
Awareness of health labeling **→** students’ attitudes **→** students’ SSB consumption	−0.196	−0.198, −0.036
Home SSB storage **→** caregivers’ SSB consumption **→** students’ SSB consumption	0.055	0.012, 0.072
Students’ knowledge level **→** students’ attitudes**→** students’ SSB consumption	−0.059	−0.116, −0.019
Health education courses **→** students’ knowledge level **→** students’ attitudes **→** students’ SSB consumption	−0.018	−0.024, −0.003

SSB, sugar-sweetened beverage; CI, confidence interval.

## 4 Discussion

The results show that 81.90% of elementary school students in Beijing consumed sugar-sweetened beverages at least once a week. This is higher than the 75.80% consumption rate among 6-18-year-olds in Shanghai (29), but lower than the 96% prevalence among 8-12-year-olds in New Zealand (30). As China’s economy grows and cities expand, dietary changes have occurred, particularly reflected in increased consumption of processed sugar and unhealthy fats. Moreover, China, along with other fast-developing countries, is also an important target market for international soft drink manufacturers. For example, sales of carbonated beverages by Coca-Cola and PepsiCo in the Chinese market more than doubled during the 2010-2020 period (31). Supporting this trend, epidemiological analyses by Guo et al. indicate that the contribution of SSBs to total daily energy intake among Chinese residents has increased significantly over the past decade (32, 33). Taken together, the high prevalence observed in this study underscores an escalating public health concern regarding SSB consumption among children in China’s megacities.

Another important result of the study was that female adolescents had a significantly higher intake of ready-to-drink beverages (e.g., pearl milk tea, fruit smoothies, and sweetened tea drinks) than male adolescents. The difference between the two sexes that we observed could be due to several reasons: for example, there are different advertising strategies for drinks aimed at appealing to girls; such beverages may be associated with social status and used to reinforce group identity among girls; or girls may be more active in social situations that involve consuming the same beverage together (34). These findings suggest that when designing public health strategies to reduce sugar-sweetened beverage intake in school-age populations, sex-specific approaches are needed.

The structural equation model in this study explains students’ SSB consumption with an R^2^ value of 0.352. According to Cohen’s effect size criteria (where *R*^2^ > 0.25 is considered a large effect) (35), this model demonstrates strong explanatory power. In terms of model fit, the χ^2^/df ratio was slightly elevated at 5.537—a common phenomenon with large sample sizes (*N* = 1,127), as the χ^2^ statistic is highly sensitive to sample size (36). Given that all other fit indices met or exceeded recommended thresholds, this deviation is unlikely to undermine the model’s validity.

### 4.1 Individual level

At the individual level, self-efficacy—defined as an individual’s confidence in their capacity to successfully execute specific health behaviors and achieve desired outcomes (37)—demonstrated a significant protective effect against SSB consumption (38). Moreover, students’ attitudes toward SSB consumption fully mediated the relationship between knowledge and consumption behavior, aligning with the knowledge–attitude–practice (KAP) model. This finding underscores that knowledge alone is insufficient to drive behavioral change; rather, it must be internalized as a favorable health attitude to exert an indirect but meaningful influence on behavior (39–42).

However, even if children develop appropriate attitudes through health education, it is difficult for them to make healthy decisions. The sweet taste of SSBs activates neural reward pathways, delivering immediate and intense sensory gratification (43). In contrast, the health benefits of reduced consumption manifest as delayed gratification, creating a temporal asymmetry between “immediate reward and delayed benefit.” This asymmetry represents a fundamental challenge in explaining why children and adolescents struggle to prioritize long-term health over immediate pleasure (44). Consequently, effective interventions should employ a dual-pathway strategy: first, strengthening children’s self-control and self-efficacy to navigate decision-making conflicts; second, offering engaging and sustainable healthy alternatives that fundamentally reduce reliance on SSBs (Supplementary Table S5).

### 4.2 Interpersonal level

Parental and peer behaviors emerged as the most significant factors promoting SSB consumption among elementary school students in this study, consistent with previous research findings (45–47). A cross-sectional study by Jaime et al. examining dietary habits and family characteristics in 4,839 families demonstrated that children in households where adults consumed SSBs daily had a significantly higher likelihood of consuming these beverages (OR = 1.78) (48). This study also found that caregivers’ and teachers’ use of SSBs as rewards increased students’ risk of consuming SSBs. This finding is in line with Bandura’s social learning theory, which states that children acquire most health-related behaviors by observing the behaviors of significant adults and caregivers (49). Consequently, interventions targeting reductions in children’s SSB consumption and related health risks—such as obesity and dental caries—achieve more favorable outcomes when they incorporate parental involvement (50).

Peer influence becomes increasingly important during late childhood and pre-adolescence (51–53). The structural equation model quantified this influence, revealing an effect of peer behavior on SSB consumption of 0.254. Notably, this effect approached that of caregivers’ behavior (*β* = 0.378), which demonstrated the strongest influence in the study. These findings suggest that during the middle and upper grades of elementary school, the exemplary influence of peers as “significant others” cannot be overlooked (54). In certain contexts, peer influence may become a core behavioral driver parallel to family influence. This finding aligns with social norms theory, which suggests that adolescents often mimic the behavior of their peers to achieve a sense of belonging and establish their identity (55). Consequently, in environments where peers congregate—such as schools and communities—interventions that deliberately promote positive health behaviors may serve as a highly valuable strategy to effectively disseminate healthy behaviors (56).

### 4.3 Environmental level

Students’ SSB consumption is driven by immediate gratification—the desire for instant sensory rewards (43). However, unlike cigarettes or alcoholic beverages, SSBs cannot be easily stockpiled or transported in bulk, rendering consumption highly dependent on proximate supply availability. Consequently, the accessibility of SSBs within children’s immediate environments—including home, school, and community settings—serves as a critical external determinant of consumption behavior. Our findings demonstrate that frequent home storage of SSBs was significantly and positively associated with increased child consumption. This observation aligns with results from a U.S. computer simulation study, which revealed that eliminating SSB availability in the home environment could reduce average weekly consumption among children aged 2–7 years by 1.23 servings, representing a 60% reduction (57). This effect substantially exceeded the 40% reduction achieved by removing SSBs exclusively from out-of-home environments such as schools and daycare centers (58).

Beyond the home environment, this study illuminated the contributions of additional environmental systems. School health education courses reduced students’ SSB consumption through the indirect pathway of KAP, whereas convenient access to community convenience stores exerted direct effects on caregivers’ SSB consumption. These findings align with Bronfenbrenner’s ecological systems theory, demonstrating that environmental systems beyond the family microsystem—including schools and communities—collectively shape children’s dietary behaviors through distinct mechanisms and pathways (58).

### 4.4 Policy recommendations

Several countries, including the United Kingdom, Chile, and Mexico, have incorporated mandatory nutrition labeling into their legislative and policy frameworks (10–12). Evidence from Chile demonstrates the effectiveness of such policies: following implementation of the Food Labeling and Advertising Law, sugar-sweetened beverage purchases declined by 23.7% (10). In China, Shanghai and Shenzhen Municipal Health Commissions have successively issued relevant norms and guidelines for health labeling on sugar-sweetened beverages (13, 14); however, policy effect evaluations based on local empirical data are currently lacking (59). Our empirical investigation of elementary school students in Beijing revealed that awareness of health labeling indirectly reduces consumption behavior by shaping students’ health attitudes toward SSBs. These findings provide critical scientific evidence to support the development and implementation of mandatory health warning label policies for SSBs in China, particularly in megacities such as Beijing.

### 4.5 Strengths and limitations

This study demonstrates several notable strengths while acknowledging important limitations. Grounded in the social-ecological model, we employed structural equation modeling to systematically quantify both direct and indirect effects of multilevel factors—including individual characteristics, interpersonal influences (caregivers’, teachers’, and peers’), environmental contexts (family, school, and community), and awareness of health labeling—on students’ SSB consumption. This comprehensive analytical approach successfully identified critical pathways, most notably revealing that caregivers’ SSB consumption exerts the strongest influence on students’ consumption patterns. These findings provide essential empirical evidence for developing targeted, multilevel intervention strategies. However, several limitations warrant consideration. First, the cross-sectional design precludes establishing causal relationships between variables; longitudinal or experimental studies are necessary to confirm temporal sequences and causality. Second, reliance on self-reported SSB consumption from students and caregivers introduces potential recall bias. Third, the overall explanatory power of the model (*R*^2^ = 0.352) was relatively low, suggesting that key predictive variables may have been omitted. We attempted to model the social-ecological influence on sugar-sweetened beverage consumption in children, but captured only a glimpse of the complex social-ecological system. Nonetheless, our findings highlight the importance of intensified collaboration between families and schools, as well as communities and public health advocates—contributing to the existing body of evidence and supporting continued efforts to reduce sugar-sweetened beverage consumption among children. Fourth, we used awareness of health labeling as a proxy to reflect the potential impact of local health labeling policies on sugar-sweetened beverage consumption. Our findings clearly point to the need for regulating such behaviors in children, which is currently lacking. However, our data are limited by the absence of additional policy-related factors—such as comprehensiveness, coerciveness, and perceived effectiveness—that reflect the multifaceted nature of health policies; therefore, policy implications drawn from the current study setting should be interpreted with caution. Future research should integrate longitudinal tracking designs with more comprehensive measurement of school and community environments to strengthen causal inference and elucidate the dynamic mechanisms underlying behavior formation.

## 5 Conclusion

This study reveals the current status, multilevel influencing factors, and key pathways of sugar-sweetened beverage consumption among elementary school students in Beijing. The results demonstrate that SSB consumption is relatively common among elementary school students, with prevalence gradually decreasing from suburban areas toward central urban areas. Structural equation modeling analysis indicates that SSB consumption is driven by multilevel factors and their interactions: at the individual level, student attitudes serve as a critical protective factor; at the interpersonal level, caregivers’ consumption exerts the strongest direct influence, followed by peer influence; at the environmental level, frequent home storage of SSBs is significantly and positively associated with increased students’ consumption, while policy factors such as awareness of health labeling primarily exert indirect effects by enhancing health attitudes. Based on these findings, this study proposes targeted intervention recommendations across individual, interpersonal, environmental, and policy levels, advocating for the construction of a comprehensive intervention system that coordinates families, schools, and communities. These findings provide important empirical evidence for precise prevention and control of childhood SSB consumption in megacities. Future research should design and implement prospective, randomized controlled intervention experiments based on the key pathways revealed in this study. Such studies will be essential to verify the scientific validity and effectiveness of the proposed intervention strategies.

## Data Availability

The original contributions presented in the study are included in the article/Supplementary material, further inquiries can be directed to the corresponding authors.
