# Supplementary material for: Factors and pathways influencing sugar-sweetened beverage consumption among children in middle childhood: a cross-sectional survey of 1,127 third-grade students in Beijing
Source: Front Public Health. 2026 May 20;14:1817212. doi: 10.3389/fpubh.2026.1817212 (PMC13229989; doi:10.3389/fpubh.2026.1817212)
Supplement: Supplementary file 1 [file Table_1.docx]

Supplementary Material

**Table S1. Associations between individual-level factors and sugar-sweetened beverage consumption.**

**Table S2. Associations between interpersonal-level factors and sugar-sweetened beverage consumption.**

**Table S3. Associations between environmental factors and sugar-sweetened beverage consumption.**

**Table S4. Associations between awareness of health labeling and sugar-sweetened beverage consumption.**

**Table S5. Multilevel intervention framework to reduce sugar-sweetened beverage consumption.**

**Table S1 Associations between individual-level factors and sugar-sweetened beverage consumption.**

| **Variable** | **Total**  **(n=1,127)** | **SSB Consumption, n (%)** | | | | **χ²/H value** | ***P*** **value** |
| --- | --- | --- | --- | --- | --- | --- | --- |
|  |  | **Never** | **1-3 times/week** | **4-6 times/week** | **≥ 7 times/week** |  |  |
| **Student’s knowledge level** |  |  |  |  |  | 3.475 | 0.062 |
| Poor | 57 | 13(22.81) | 7(12.28) | 6(10.53) | 31(54.39) |  |  |
| Needs improvement | 108 | 19(17.59) | 17(15.74) | 12(11.11) | 60(55.56) |  |  |
| Good | 962 | 172(17.88) | 240(24.95) | 153(15.90) | 397(41.27) |  |  |
| **Health beliefs** |  |  |  |  |  | 1.874 | 0.171 |
| Poor | 443 | 76(17.16) | 104(23.48) | 72(16.25) | 191(43.12) |  |  |
| Good | 684 | 128(18.71) | 160(23.39) | 99(14.47) | 297(43.42) |  |  |
| **Self-efficacy** |  |  |  |  |  | 5.655 | 0.017 |
| Poor | 444 | 61(13.74) | 81(18.24) | 66(14.86) | 236(53.15) |  |  |
| Good | 683 | 143(20.94) | 183(26.79) | 105(15.37) | 252(36.90) |  |  |
| **Emotional attitudes** |  |  |  |  |  | 9.288 | 0.002 |
| Poor | 526 | 85(16.16) | 95(18.06) | 84(15.97) | 262(49.81) |  |  |
| Good | 601 | 119(19.80) | 169(28.12) | 87(14.48) | 226(37.60) |  |  |

SSB, sugar-sweetened beverage.

**Table S2 Associations between interpersonal-level factors and sugar-sweetened beverage consumption.**

| **Variable** | **Total**  **(n=1,127)** | **SSB Consumption, n (%)** | | | | **χ²** | ***P*** **value** |
| --- | --- | --- | --- | --- | --- | --- | --- |
|  |  | **Never** | **1-3 times/week** | **4-6 times/week** | **≥ 7 times/week** |  |  |
| **Caregivers’ knowledge level** |  |  |  |  |  | 5.017 | 0.025 |
| Poor | 2 | 2(100.00) | 0(0.00) | 0(0.00) | 0(0.00) |  |  |
| Needs improvement | 46 | 12(26.09) | 14(30.43) | 1(2.17) | 19(41.30) |  |  |
| Good | 1079 | 190(17.61) | 250(23.17) | 170(15.76) | 469(43.47) |  |  |
| **Caregivers’ attitudes toward restricting students’ SSBs** |  |  |  |  |  | 0.182 | 0.670 |
| Strongly Disagree | 28 | 8(28.57) | 9(32.14) | 3(10.71) | 8(28.57) |  |  |
| Disagree | 31 | 2(6.45) | 8(25.81) | 4(12.90) | 17(54.84) |  |  |
| Neither Agree nor Disagree | 130 | 19(14.62) | 19(14.62) | 30(23.08) | 62(47.69) |  |  |
| Agree | 234 | 41(17.52) | 64(27.35) | 38(16.24) | 91(38.89) |  |  |
| Strongly Agree | 704 | 134(19.03) | 164(23.30) | 96(13.64) | 310(44.03) |  |  |
| **Caregivers’ attitudes toward restricting students’ fried foods** |  |  |  |  |  | 0.003 | 0.954 |
| Strongly Disagree | 20 | 3(15.00) | 6(30.00) | 2(10.00) | 9(45.00) |  |  |
| Disagree | 31 | 3(9.68) | 14(45.16) | 3(9.68) | 11(35.48) |  |  |
| Neither Agree nor Disagree | 225 | 43(19.11) | 42(18.67) | 40(17.78) | 100(44.44) |  |  |
| Agree | 313 | 53(16.93) | 77(24.60) | 60(19.17) | 123(39.30) |  |  |
| Strongly Agree | 538 | 102(18.96) | 125(23.23) | 66(12.27) | 245(45.54) |  |  |
| **Caregivers’ attitudes toward restricting students’ high-sugar snacks** |  |  |  |  |  | 2.184 | 0.139 |
| Strongly Disagree | 27 | 7(25.93) | 7(25.93) | 2(7.41) | 11(40.74) |  |  |
| Disagree | 18 | 1(5.56) | 8(44.44) | 2(11.11) | 7(38.89) |  |  |
| Neither Agree nor Disagree | 125 | 27(21.60) | 19(15.20) | 24(19.20) | 55(44.00) |  |  |
| Agree | 298 | 57(19.13) | 81(27.18) | 49(16.44) | 111(37.25) |  |  |
| Strongly Agree | 659 | 112(17.00) | 149(22.61) | 94(14.26) | 304(46.13) |  |  |
| **Caregivers’ SSB consumption** |  |  |  |  |  | 61.894 | <0.001 |
| Never | 125 | 47(37.60) | 23(18.40) | 12(9.60) | 43(34.40) |  |  |
| 1-3 times/week | 208 | 47(22.60) | 78(37.50) | 19(9.13) | 64(30.77) |  |  |
| 4-6 times/week | 165 | 35(21.21) | 41(24.85) | 29(17.58) | 60(36.36) |  |  |
| ≥ 7 times/week | 629 | 75(11.92) | 122(19.40) | 111(17.65) | 321(51.03) |  |  |
| **Caregivers’ rewards^a^** |  |  |  |  |  | 10.105 | 0.001 |
| Never | 704 | 139(19.74) | 179(25.43) | 97(13.78) | 289(41.05) |  |  |
| Occasionally | 381 | 64(16.80) | 78(20.47) | 69(18.11) | 170(44.62) |  |  |
| Often | 32 | 1(3.13) | 5(15.63) | 5(15.63) | 21(65.63) |  |  |
| **Teachers’ rewards^a^** |  |  |  |  |  | 6.044 | 0.014 |
| Never | 1032 | 193(18.70) | 242(23.45) | 166(16.09) | 431(41.76) |  |  |
| Occasionally | 73 | 10(13.70) | 19(26.03) | 5(6.85) | 39(53.42) |  |  |
| Often | 12 | 1(8.33) | 1(8.33) | 0(0.00) | 10(83.33) |  |  |
| **Peers’ SSB consumption^b^** |  |  |  |  |  | 20.466 | <0.001 |
| Never | 174 | 41(23.56) | 36(20.69) | 27(15.52) | 70(40.23) |  |  |
| Occasionally | 701 | 132(18.83) | 190(27.10) | 114(16.26) | 265(37.80) |  |  |
| Often | 240 | 31(12.92) | 35(14.58) | 29(12.08) | 145(60.42) |  |  |
| **Peer-influenced SSB purchase^c^** |  |  |  |  |  | 66.949 | <0.001 |
| Never | 474 | 116(24.47) | 127(26.79) | 58(12.24) | 173(36.50) |  |  |
| Occasionally | 525 | 83(15.81) | 131(24.95) | 103(19.62) | 208(39.62) |  |  |
| Often | 117 | 5(4.27) | 4(3.42) | 10(8.55) | 98(83.76) |  |  |
| **SSB sharing with peers^d^** |  |  |  |  |  | 46.810 | <0.001 |
| Never | 479 | 114(23.80) | 115(24.01) | 72(15.03) | 178(37.16) |  |  |
| Occasionally | 458 | 76(16.59) | 125(27.29) | 87(19.00) | 170(37.12) |  |  |
| Often | 177 | 14(7.91) | 22(12.43) | 12(6.78) | 129(72.88) |  |  |

a, missing 10; b, missing 12; c, missing 11; d, missing 13; SSB, sugar-sweetened beverage.

**Table S3 Associations between environmental factors and sugar-sweetened beverage consumption.**

| **Variable** | **Total**  **(n=1,127)** | **SSB Consumption n (%)** | | | | **χ²** | ***P*** **value** |
| --- | --- | --- | --- | --- | --- | --- | --- |
|  |  | **Never** | **1-3 times/week** | **4-6 times/week** | **≥ 7 times/week** |  |  |
| **Frequency of SSB storage in the home^a^** |  |  |  |  |  | 49.150 | <0.001 |
| None | 427 | 100(23.42) | 111(26.00) | 65(15.22) | 151(35.36) |  |  |
| Occasionally | 516 | 91(17.64) | 129(25.00) | 88(17.05) | 208(40.31) |  |  |
| Frequently | 175 | 13(7.43) | 22(12.57) | 18(10.29) | 122(69.71) |  |  |
| **Supermarket convenience^b^** |  |  |  |  |  | 0.104 | 0.747 |
| Yes, convenient | 902 | 167(18.51) | 211(23.39) | 133(14.75) | 391(43.35) |  |  |
| Yes, inconvenient | 94 | 14(14.89) | 23(24.47) | 21(22.34) | 36(38.30) |  |  |
| None | 126 | 23(18.25) | 29(23.02) | 16(12.70) | 58(46.03) |  |  |
| **Convenience store access^c^** |  |  |  |  |  | 5.792 | 0.016 |
| Yes, convenient | 1008 | 175(17.36) | 232(23.02) | 156(15.48) | 445(44.15) |  |  |
| Yes, inconvenient | 72 | 17(23.61) | 21(29.17) | 9(12.50) | 25(34.72) |  |  |
| None | 40 | 11(27.50) | 10(25.00) | 5(12.50) | 14(35.00) |  |  |
| **Beverage vending machine convenience^d^** |  |  |  |  |  | 0.286 | 0.593 |
| Yes, convenient | 605 | 108(17.85) | 137(22.64) | 94(15.54) | 266(43.97) |  |  |
| Yes, inconvenient | 98 | 19(19.39) | 27(27.55) | 15(15.31) | 37(37.76) |  |  |
| None | 407 | 75(18.43) | 98(24.08) | 59(14.50) | 175(43.00) |  |  |
| **Traditional market convenience^e^** |  |  |  |  |  | 0.443 | 0.506 |
| Yes, convenient | 771 | 144(18.68) | 184(23.87) | 114(14.79) | 329(42.67) |  |  |
| Yes, inconvenient | 130 | 24(18.46) | 28(21.54) | 21(16.15) | 57(43.85) |  |  |
| None | 210 | 35(16.67) | 49(23.33) | 34(16.19) | 92(43.81) |  |  |
| **Restaurant convenience^f^** |  |  |  |  |  | 0.558 | 0.455 |
| Yes, convenient | 936 | 179(19.12) | 218(23.29) | 137(14.64) | 402(42.95) |  |  |
| Yes, inconvenient | 120 | 12(10.00) | 32(26.67) | 20(16.67) | 56(46.67) |  |  |
| None | 57 | 12(21.05) | 11(19.30) | 11(19.30) | 23(40.35) |  |  |
| **Frequency of health education courses^g^** |  |  |  |  |  | 0.157 | 0.692 |
| None | 506 | 99(19.57) | 111(21.94) | 82(16.21) | 214(42.29) |  |  |
| Occasionally | 524 | 87(16.60) | 132(25.19) | 79(15.08) | 226(43.13) |  |  |
| Frequently | 85 | 17(20.00) | 19(22.35) | 10(11.76) | 39(45.88) |  |  |

a, 9 missing; b, 5 missing; c, 7 missing; d, 17 missing; e, 16 missing; f, 14 missing; g, 12 missing; SSB, sugar-sweetened beverage.

**Table S4 Associations between** **awareness of health labeling and sugar-sweetened beverage consumption.**

| **Variable** | **Total**  **(n=1,127)** | **SSB Consumption n (%)** | | | | **χ²/H value** | ***P*** **value** |
| --- | --- | --- | --- | --- | --- | --- | --- |
|  |  | **Never** | **1-3 times/week** | **4-6 times/week** | **≥ 7 times/week** |  |  |
| **Impact on SSB purchase^a^** |  |  |  |  |  | 42.029 | <0.001 |
| Buy more | 29 | 5(17.24) | 7(24.14) | 1(3.45) | 16(55.17) |  |  |
| No change | 157 | 21(13.38) | 23(14.65) | 12(7.64) | 101(64.33) |  |  |
| Buy less | 308 | 38(12.34) | 70(22.73) | 60(19.48) | 140(45.45) |  |  |
| Choose low‑/no‑sugar beverages | 367 | 67(18.26) | 102(27.79) | 51(13.90) | 147(40.05) |  |  |
| Give up purchasing | 256 | 73(28.52) | 60(23.44) | 47(18.36) | 76(29.69) |  |  |
| **Discourage family members/friends from consuming SSB^b^** |  |  |  |  |  | 48.124 | <0.001 |
| Yes | 711 | 151(21.24) | 195(27.43) | 119(16.74) | 246(34.60) |  |  |
| No | 397 | 52(13.10) | 67(16.88) | 51(12.85) | 227(57.18) |  |  |
| **Develop healthy eating habits^c^** |  |  |  |  |  | 27.176 | <0.001 |
| Very meaningful | 516 | 120(23.26) | 144(27.91) | 81(15.70) | 171(33.14) |  |  |
| Meaningful | 383 | 48(12.53) | 82(21.41) | 56(14.62) | 197(51.44) |  |  |
| Slightly meaningful | 136 | 23(16.91) | 21(15.44) | 27(19.85) | 65(47.79) |  |  |
| Not meaningful | 79 | 12(15.19) | 15(18.99) | 7(8.86) | 45(56.96) |  |  |

a, 10 missing; b, 19 missing; c, 13 missing; SSB, sugar-sweetened beverage.

**Table S5** **Multilevel intervention framework to reduce sugar-sweetened beverage consumption.**

| **Intervention Level** | **Intervention Target** | **Recommended Intervention Measures** |
| --- | --- | --- |
| Individual | Students | 1. Enhance self-efficacy for healthy behaviors: Use experiential activities (e.g., "Healthy Challenge Week") to help students successfully reduce SSB consumption and strengthen their confidence.  2. Transform knowledge into positive attitudes: Design interactive courses to promote students' internalization of health beliefs.  3. Provide attractive healthy alternatives and exercise prescriptions: Promote delicious, healthy beverages paired with fun, easy-to-participate exercise programs to offset the sensory rewards of sugar-sweetened beverages with immediate health rewards. |
| Interpersonal | Caregivers | Promote behavior change and participation: Guide parents to become healthy role models (e.g., family "beverage-free days") and to learn how to read food labels. |
|  | Peers | Establish and utilize positive peer role models: Recruit and train “health ambassadors” in schools and communities to shape healthy group norms through peer education, group competitions, and other methods. |
| Environmental | Home environment | Reduce household accessibility to unhealthy beverages: Use science education and practical guidance to help families develop shopping lists, conduct inventory checks, and replace sugar-sweetened beverages with healthy drinks (e.g., milk and plain water). |
|  | School and community environment | 1. Strengthen the school health education curriculum: Collect and curate evidence-informed health education lesson plans to systematically integrate nutrition knowledge and critical thinking skills into existing curricula.  2. Limit community accessibility to unhealthy beverages: Promote “no high-sugar beverage sales” campaigns within a specified radius of school perimeters (e.g., 200–500 meters) or encourage convenience stores to establish dedicated healthy beverage sections. |

SSB, sugar-sweetened beverage.
